# Acupuncture for post-stroke insomnia: A protocol for systematic review and meta-analysis

**DOI:** 10.1097/MD.0000000000021381

**Published:** 2020-07-24

**Authors:** Jie Xiang, Honglian Li, Jun Xiong, Fanghui Hua, Shouqiang Huang, Yunfeng Jiang, Xiaohong Zhou, Kai Liao, Lingling Xu

**Affiliations:** a Jiangxi University of Traditional Chinese Medicine; b Haiyang People's Hospital of Shandong Province, Haiyang; c Affiliated Hospital of Jiangxi University of Traditional Chinese Medicine, Nanchang, P.R. China.

**Keywords:** acupuncture, post-stroke insomnia, protocol, systematic review

## Abstract

**Background:**

Post-stroke insomnia (PSI) is a significant complication of stroke, which often affects patients in various aspects. Acupuncture has fewer side effect and is increasingly used to treat PSI. The purpose of this study is to summarize the efficacy and safety of acupuncture for PSI.

**Methods:**

We will perform a comprehensive electronic searching, including PubMed, Embase, Cochrane Library, WangFang Database, China National Knowledge Infrastructure, Chinese Scientific Journal Database, Chinese Biomedical Literature Database, from inception to July 2020. We will also manually retrieve references, and contact lead authors. Randomized clinical trials (RCTs) of acupuncture for PSI will be included, regardless of whether blind method and allocation concealment are used. The outcomes of interest include: Pittsburgh Sleep Quality Index (PSQI), Insomnia Severity Index (ISI), efficacy standards of Chinese medicine, relapse rate after follow-up, adverse events, quality of life. To assess the risk of bias, we will use the Cochrane risk assessment tool. RevMan 5.3 software will be used to conduct data synthesis. The evidence quality of each outcome will be appraised according to Grades of Recommendation, Assessment, Development, and Evaluation (GRADE).

**Results:**

The results will be published in a peer-reviewed journal.

**Conclusion:**

This study will provide a high-quality evidence to evaluate the efficacy and adverse reactions of acupuncture for PSI.

**PROSPERO registration number:**

CRD42020157865.

## 1 Introduction

Stroke is a major fatal disease in the world, and it is also a leading cause of disability in adults, with a high risk of relapse.^[1]^ Sleep disorders is a common complication of stroke, of which the most common type is insomnia.^[2]^ The main manifestations of insomnia are difficulty falling asleep and maintaining sleep, early morning awakening, and nonrestorative sleep.^[3]^ The incidence of post-stroke insomnia (PSI) is different in different studies. A related meta-analysis shows that the incidence is as high as 38.2%.^[4]^ Insomnia not only affects stroke recovery and quality of life, but also increases the risk of stroke recurrence and mental disorders such as anxiety and cognitive decline.^[5–8]^ The pathogenesis of PSI is complex, and the research is insufficient. Current studies have shown that it may be related to the location of stroke, imbalance of neurotransmitters, changes of inflammatory factors, and psychosocial factors.^[9–12]^

Western medicine treatment of PSI mainly includes benzodiazepines, non-benzodiazepines, melatonin receptor agonists, such as alprazolam, estazolam, diazepam, zolpidem tartrate, dexzopiclone, zaleplon, Remelton, agomelatine.^[13]^ Although these drugs are effective, we should not ignore their side effects, such as drug dependence, drowsiness, dizziness, and cognitive impairment.^[14,15]^ As a result, many people seek alternative therapies, most commonly cognitive behavioral therapy, which, according to one study, has better long-term efficacy than western drugs, but less short-term efficacy than drugs.^[16]^ A increasing number of patients are being treated with acupuncture, and recently the frequency of visits by acupuncturists in the United States has increased significantly.^[17]^ Acupuncture has been shown in several studies to be effective in treating insomnia.^[18–20]^

Acupuncture is a treatment of traditional Chinese medicine (TCM). Acupuncture is a kind of mechanical stimulation, the essence of the effect is to stimulate and promote the body's own adjustment function and self-rehabilitation ability to treat diseases, the advantage of acupuncture is that there are no side effects of drugs.^[21]^

According to TCM theory, the causes of PSI are deficiency of vital qi, failure of yang to enter yin, internal injury of fatigue, damage of 5 internal organs, loss of health and movement of spleen, internal growth of turbid phlegm, injury of 5 minds, excessive 7 emotions, exogenous evil qi, and disharmony between Ding.^[22]^ Acupuncture has the function of dredging channels and collaterals, coordinating yin and yang, and adjusting viscera, can stimulate patients’ own meridian qi to resist evil, and rebalance human qi and blood yin and yang. According to the relevant experimental research results, the main mechanism of acupuncture in the treatment of PSI may be related to reducing the damage of awakening functional areas and regulating neurotransmitters and cytokines related to awakening.^[23]^

Currently, there are 4 systematic reviews and meta-analysis of acupuncture for PSI, 3 of which are in Chinese and 1 is in English.^[24–27]^ But they have some defects, such as insufficient search, unregistered, few outcome indicators, lack of evidence quality evaluation, and long publication time. Therefore, there is an urgent need to update and improve the systematic review and meta-analysis of acupuncture of PSI to obtain high-quality evidence-based evidence and better guide clinical practice. We will perform this systematic review according to the latest resources in the hope that provide reliable evidence and establish a better approach for treating PSI with acupuncture.

## 2 Methods

### 2.1 Study registration

The protocol has been registered on PROSPERO (no: CRD42020157865. https://www.crd.york.ac.uk/PROSPERO/display_record.php?RecordID=157865). We will complete this protocol according to the Preferred Reporting Items for Systematic reviews and Meta-Analysis Protocols (PRISMA-P).^[28]^ The changes will be described in our full review if needed.

### 2.2 Inclusion criteria

#### 2.2.1 Type of studies

We will only need randomized clinical trials (RCTs) about acupuncture for PSI, regardless of whether blind method and allocation concealment are used.

#### 2.2.2 Type of participants

The patients in the study will be those who are diagnosed with PSI at the age of 18 or older. There will be no restrictions on diagnostic criteria, race, sex, economic situation, and education level.

#### 2.2.3 Type of interventions

Different types of acupuncture interventions will be included (e.g., manual acupuncture, ear acupuncture, electroacupuncture, etc). Single acupuncture treatment and acupuncture as the main part of the combined therapy will be included. Highly heterogeneous interventions will be excluded, such as laser acupuncture, cupping, acupoint catgut embedding, Chinese Tuina, and Chinese herbs.

#### 2.2.4 Type of comparators

The control interventions will be no treatment or sham acupuncture, placebo, and other active interventions. Eligible comparisons include acupuncture versus no treatment, acupuncture versus placebo or sham acupuncture, acupuncture versus other active therapies, acupuncture + active therapy versus the same active therapy.

#### 2.2.5 Types of outcome measures

##### 2.2.5.1 Primary outcomes

The Pittsburgh Sleep Quality Index (PSQI).

##### 2.2.5.2 Secondary outcomes

1. The Insomnia Severity Index (ISI).
2. The efficacy standards of Chinese medicine.
3. Relapse rate after follow-up.
4. Adverse events.
5. Quality of life.

### 2.3 Exclusion criteria

We will exclude repeated publications and full text cannot be obtained through various sources.

### 2.4 Search methods for identification of studies

#### 2.4.1 Data sources

We will perform a comprehensive electronic searching, including PubMed, Embase, Cochrane Library, WangFang Database, China National Knowledge Infrastructure, Chinese Scientific Journal Database, Chinese Biomedical Literature Database, from inception to July 2020. We will also manually retrieve references, and contact lead authors.

#### 2.4.2 Search strategy

MeSH and text words will be included in the search fields. Publication language will be unrestricted. The search strategy for PubMed is shown in Table 1.

**Table 1:**
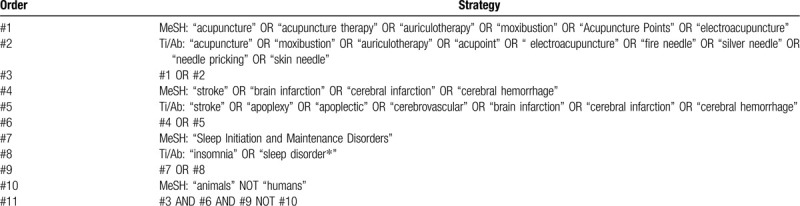
The search strategy for PubMed.

### 2.5 Studies selection

**Figure 1:**
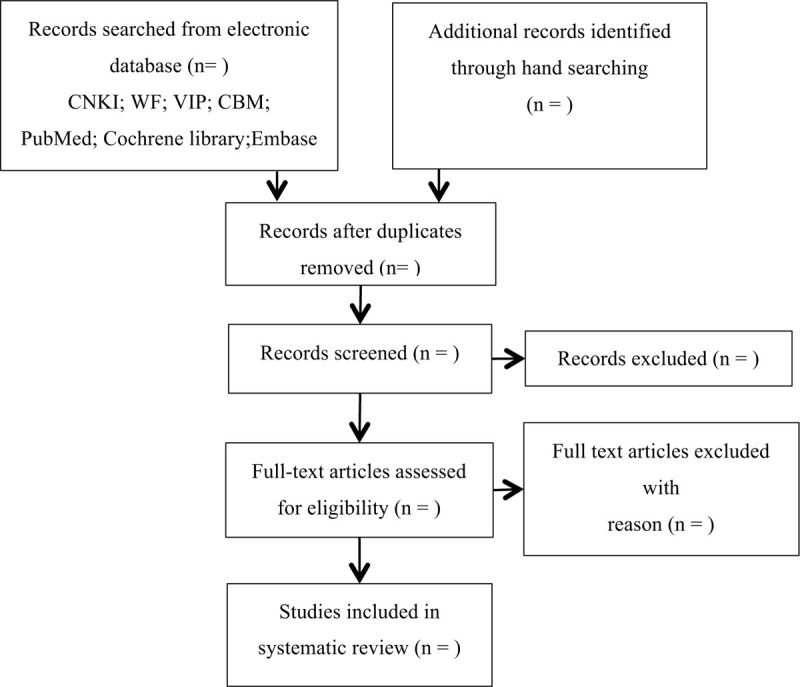
Flowchart of literature selection.

We will import the retrieved studies into NoteExpress 3.2.0.7535 and discard the duplicated studies. Two reviewers (JX and FH) will exclude the literatures that do not meet the inclusion criteria by reading the titles and abstracts. Then, the reviewers will check the full texts to determine final decision according to the criteria. All the screening processes will be conducted independently. If the articles information is insufficient, we will try to contact the authors to obtain the necessary details. When 2 reviewers have different opinions, the final decision will be made by the third reviewer (SH). The flow process of selection is shown in a PRISMA flow chart (Fig. 1).

### 2.6 Data extraction

The data will be extracted independently by 2 reviewers (YJ and XZ), including the following information: author, publish year, participant characteristics, intervention (s), comparison (s), outcome (s), and some relevant characteristics. Any disagreements will be decided through discussion or the third reviewer (KL).

### 2.7 Data analysis

#### 2.7.1 Assessment of risk of bias and reporting quality of studies

To assess the risk of bias, we will use the Cochrane risk assessment tool.^[29]^ The following 7 items will be included: random sequence generation, allocation concealment, blinding of participants and caregivers, blinding of outcome evaluator, incomplete outcome data, selective reporting, and other bias. High, low, unclear assessments will be performed for each item. Meanwhile, the Standards for Reporting Interventions in Clinical Trials of Acupuncture checklist will be also completed.^[30]^ Disagreement will be solved by communication or consultation with a third reviewer (JX).

#### 2.7.2 Measures of treatment effect

We will use mean difference (MD) or standard mean difference (SMD) with 95% confidence intervals (CIs) as the effect measure for continuous data. The dichotomous outcomes will be analyzed by risk ratio (RR) with 95% CIs.

#### 2.7.3 Dealing with missing data

When there are events in the reports that are unclear or do not report data, we will contact the author by phone or email to obtain complete information.

#### 2.7.4 Assessment of heterogeneity

We will use RevMan5.3.5 software (Copenhagen: The Nordic Cochrane Centre, The Cochrane Collaboration, 2014) to detect the heterogeneity between studies.^[31,32]^ When *P* < .1, *I*^2^ > 50%, there is significant heterogeneity between studies; otherwise, heterogeneity is acceptable.

#### 2.7.5 Data synthesis

RevMan5.3.5 will be used for all statistical analyses. We will use the random effects model to merge the data. The results of the meta-analyses will be presented by forest plots. When the results are unsuitable to be combined due to the clinical or methodologic heterogeneity, we will perform descriptive analysis.

#### 2.7.6 Subgroup analysis

If necessary, subgroup analyses will be performed according to the different types of participant characteristics, acupuncture therapies, treatment frequency.

#### 2.7.7 Sensitivity analysis

If the result shows high heterogeneity (the *I*^2^ test is >75%), we will conduct sensitivity analysis. Then we will acquire a stable result of our study.

#### 2.7.8 Publication bias

When the included studies >10, we will use the funnel plot to assess publication bias.

#### 2.7.9 Summary of evidence

The evidence quality of each outcome will be evaluated according to Grades of Recommendation, Assessment, Development, and Evaluation (GRADE).^[33]^ This process will be carried out independently by the 2 reviewers (HL and KL), and if there are differences, the third author (JX) will decide.

#### 2.7.10 Ethics and dissemination

In this study, no individual data from participants will be involved, so ethics approval is not required. This systematic review will be published through peer-reviewed journal.

## 3 Discussion

PSI is a common complication of stroke, which often affects patients in various aspects.^[34]^ It is also a risk factor for some chronic diseases (e.g., hypertension, diabetes).^[35]^ Therefore, it is necessary to intervene in PSI.

Acupuncture is a treatment with quick curative effect and little side effect. Acupuncture can resist the damage of free radicals, regulate cellular Ca^2+^ homeostasis, antagonize the release of excitatory amino acids, improve cerebral hemodynamics, regulate monoamine neurotransmitters, and regulate cytokines.^[23]^ Based on the syndrome differentiation and treatment of TCM, we can choose different types of acupuncture method, choose different acupoints to treat PSI. In recent years, an increasing number of researchers have conducted clinical trials of acupuncture for PSI. Therefore, it is necessary to conduct a systematic review to establish convincing evidence to prove the effectiveness and safety of acupuncture for PSI. Due to the limitations of previous systematic reviews, such as unregistered, insufficient search, few outcome indicators, long publication time, and lack of evidence quality evaluation, the results may be uncertain. Therefore, we will adopt a more rigorous method for systematic review, hoping to provide reliable evidence for the clinical decision.

However, there may be some potential limitations in this systematic evaluation. First of all, due to different acupuncture methods, heterogeneity may be greater. Secondly, the quality of RCTs may be low and there is a risk of bias.

## Author contributions

**Conceptualization:** Jie Xiang, Jun Xiong.

**Data curation:** Jie Xiang, Fanghui Hua, Shouqiang Huang.

**Formal analysis:** Yunfeng Jiang, Xiaohong Zhou, Lingling Xu.

**Investigation:** Jun Xiong, Jie Xiang.

**Methodology:** Jun Xiong, Fanghui Hua, Shouqiang Huang, Honglian Li.

**Software:** Yunfeng Jiang, Xiaohong Zhou, Lingling Xu.

**Supervision:** Jun Xiong, Kai Liao.

**Writing – original draft:** Jie Xiang, Jun Xiong, Fanghui Hua, Shouqiang Huang.

**Writing – review & editing:** Honglian Li, Yunfeng Jiang, Xiaohong Zhou, Kai Liao, Lingling Xu.
